# Synergistic Effect of Low-Intensity Ultrasound and Co-Culture Lactic Acid Bacteria Fermentation on the Antioxidant Properties of *Echium amoenum*

**DOI:** 10.3390/antiox15030335

**Published:** 2026-03-06

**Authors:** Ehsan Divan Khosroshahi, Rita Abi Rached, Maria Manconi, Maria Letizia Manca, Mohammad Firoznezhad, Mansureh Ghavam, Seyed Hadi Razavi, Zeinab E. Mousavi, Maryam Salami

**Affiliations:** 1 Bioprocess Engineering Laboratory (BPEL), Department of Food Science and Engineering, Faculty of Agricultural Engineering and Technology, University of Tehran, Karaj 31587-77871, Iran; ehsan.khosroshahi@ut.ac.ir (E.D.K.); msalami@ut.ac.ir (M.S.); 2 Department of Life and Environmental Sciences, University of Cagliari, University Campus, Pad. A, S.P. Monserrato-Sestu Km 0.700, 09042 Monserrato, Italy; rita.abirached@unica.it (R.A.R.); manconi@unica.it (M.M.); 3 Fondazione Edmund Mach, Istituto Agrario San Michele All’Adige, Via E. Mach, 1, 38098 S. Michele all’Adige, Italy; m_firoznejhad_20500@yahoo.com; 4 Department of Nature Engineering, Faculty of Natural Resources and Earth Sciences, University of Kashan, Kashan 87317-53153, Iran; mansurehghavam@gmail.com; 5 Functional Food Research Core, University of Tehran, Tehran 14179-35840, Iran; 6 Bioprocessing and Biodetection Laboratory, Department of Food Science and Engineering, Campus of Agriculture and Natural Resources, University of Tehran, Karaj 31587-77871, Iran; zeinab.mosavi@ut.ac.ir

**Keywords:** *Echium amoenum*, low-intensity ultrasound, co-culture fermentation, lactic acid bacteria (LAB), phenolic compounds, flavonoids, antioxidant potential

## Abstract

This study investigates the dynamic physicochemical and microbiological changes during low-intensity ultrasound-assisted co-culture lactic acid bacteria (LAB) fermentation (*Lactobacillus acidophilus*, *Lactiplantibacillus plantarum*, and *Limosilactobacillus reuteri*) of *Echium amoenum* over 24 h. The pH decreased from an initial 6.5 to 3.9 after fermentation, coinciding with a peak bacterial biomass of 6.8 CFU/mL, demonstrating robust microbial activity and growth. In the unfermented sample, the maximum phenolics (147.23 ± 0.17 mg of gallic acid equivalents (GAE)/g of dry weight (DW)) and flavonoids (53.11 ± 0.41 mg of quercetin (QE)/g DW) amounts, along with antioxidant activity measured by means of 2,2-diphenyl-1-picrylhydrazyl (DPPH, 35.08 ± 1.56 mg GAE/g DW), and ferric reducing antioxidant power (FRAP, 1.02 ± 0.05 mmol FeSO_4_ equivalents/g DW) were reached at 24 h. In contrast, the ultrasound-assisted co-culture LAB fermentation using *L. acidophilus* and *L. plantarum* exhibited the highest phenolic (299.42 ± 0.89 mg GAE/g DW) and flavonoid (88.39 ± 1.53 mg QE/g DW) values, as well as the highest antioxidant activity (DPPH 64.02 ± 1.67 mg GAE/g DW and FRAP 1.96 ± 0.01 mmol FeSO_4_ equivalents/g DW), after 12 h of fermentation, indicating enhanced extraction efficiency, metabolite biosynthesis, and reduced processing time. Furthermore, FTIR analysis confirmed structural alterations in polyphenolic functional groups. Low-intensity ultrasound and co-cultured LAB fermentation synergistically accelerated microbial growth, bioactive compound release, and antioxidant capacity in *E. amoenum*, highlighting the efficiency of bioprocessing for functional foods and nutraceuticals.

## 1. Introduction

*Echium amoenum*, a highly valued medicinal plant in Iran, is known for its rich polyphenolic profile and strong antioxidant activity [1,2,3,4,5]. However, the bioavailability of plant-derived phenolic compounds is generally low (approximately 5–10%), prompting the use of microbial fermentation as an effective strategy to enhance their release and bioavailability through the liberation of bound phenolics from the plant cell wall matrix. In this context, lactic acid bacteria (LAB) fermentation has been widely recognized as an efficient bioprocess for improving the release and biosynthesis of functional metabolites [6]. In addition, ultrasound treatment, considered a green and innovative technology [7], can further enhance fermentation efficiency by stimulating microbial activity [8,9,10] and facilitating the release of conjugated phenolic compounds through increased membrane permeability, improved mass transfer, and greater substrate accessibility [11,12].

Species of the genus *Echium* (Boraginaceae) are distributed across the Macaronesian Islands, the Mediterranean Basin, and the Irano-Turanian region, and are characterized by distinctive purple or blue flowers [1,3,4]. Among them, *E. amoenum* has a long history of traditional use, commonly consumed as decoctions for its tonic, soothing, and antitussive effects [2]. Owing to its broad spectrum of biological activities, this species has gained considerable interest in the pharmaceutical, food, and cosmetic industries [7]. Studies have shown that dried *E. amoenum* petals exhibit antioxidant, anti-inflammatory, anxiolytic, antiviral, and antidepressant activities, which are mainly attributed to their complex phytochemical composition, including phenolic acids, flavonoids, carotenoids, and anthocyanins [2,4,12].

Microbial fermentation, as an economic and natural process, has traditionally been used for various applications and stands as a forerunner in modern biotechnology [13]. Its capacity to convert raw and waste materials into valuable end-products through microbial action has significantly impacted multiple sectors [7,13]. Microbiological fermentation leverages microorganisms to modify and enhance the bioactive potential of beneficial products. By breaking down plant cell walls and promoting hydrolysis, fermentation increases the content of polyphenols, flavonoids, organic acids, proteins, ceramides, amino acids, biological enzymes, and antioxidants in raw plant resources [7,14,15]. More specifically, microorganisms secrete enzymes, including cellulases and pectinases, which may improve the permeability or even degrade plant cell walls, thereby improving the liberation of active molecules. Furthermore, glycosyl hydrolases, phenolic acid decarboxylases, and reductases mediate the biotransformation of glycosides, phenolic acids, and proteins into bioactive metabolites, enhancing the nutritional and pharmacological properties. The production of antioxidant enzymes, such as superoxide dismutase and catalase, by microorganisms during fermentation further potentiates the antioxidant capacity. Consequently, fermentation represents a valuable technique for processing natural pharmaceuticals [7,16]. In particular, LAB fermentation demonstrably improves the functional attributes of plant-based products, enhancing their antioxidant capacity, anti-aging properties, and other health-promoting effects [7,13,17]. The resultant functional blended fermented beverage utilizing edible rose and shiitake mushroom with five commercial LAB strains demonstrated increased levels of total phenols, total flavonoids, and free amino acids, along with enhanced antioxidant activity [18]. Furthermore, co-fermentation of wolfberry–longan juice using *Lacticaseibacillus paracasei* ZH8 and *Lactococcus lactis* subsp. *lactis* YM313 notably improved total phenolic and flavonoid content, thereby enhancing antioxidant properties [19]. Based on this, *Lactobacillus acidophilus*, *Lactiplantibacillus plantarum*, and *Limosilactobacillus reuteri* were selected based on their proven adaptability to plant-based substrates, tolerance to acidic environments, and ability to produce enzymes involved in the biotransformation of phenolic compounds. In particular, *L. plantarum* is well known for its phenolic acid decarboxylase and reductase activities, while *L. acidophilus* and *L. reuteri* contribute to organic acid production and redox modulation. The co-culture strategy was adopted to exploit potential metabolic complementarity and synergistic interactions among strains, which have been reported to enhance bioactive compound release and antioxidant capacity compared to single-strain fermentations [20].

However, conventional plant fermentation presents several limitations, including long processing times, limited efficiency, incomplete substrate degradation, restricted nutrient release, and inconsistent environmental control, often resulting in reduced metabolite yields. To overcome these constraints, ultrasound technology has been increasingly employed to improve the efficiency, controllability, and scalability of fermentation processes while reducing environmental impact and safety concerns [21]. When integrated with fermentation, ultrasound enhances the release of bioactive compounds, such as phenolics and flavonoids, by disrupting plant cell walls and promoting enzyme activity. In addition, it improves mass transfer, mixing, and homogenization, thereby facilitating nutrient and gas diffusion within the fermentation medium [21]. Ultrasound pretreatment can also stimulate microbial metabolism, cell growth, enzyme production, and metabolite synthesis. Notably, low-intensity ultrasound (20–100 kHz) has been shown to transiently increase microbial cell membrane permeability, enhancing substrate uptake, extracellular enzyme secretion, and overall fermentation performance, thus highlighting its potential for industrial fermentation applications [21].

Despite its potential advantages, ultrasound treatment may also present limitations if improperly applied. High-intensity or prolonged ultrasound exposure has been reported to induce microbial cell damage, excessive cavitation, free radical generation, and degradation of thermolabile or oxidation-sensitive bioactive compounds. Such effects may negatively impact fermentation performance and antioxidant stability [22,23]. Therefore, careful optimization of ultrasound parameters, particularly intensity, frequency, and exposure duration, is essential to ensure beneficial rather than detrimental outcomes.

This study aims to elucidate the synergistic effect of low-intensity ultrasound and co-culture LAB fermentation on physicochemical properties, focusing on phenolic and flavonoid enrichment, antioxidant capacity (2,2-diphenyl-1-picrylhydrazyl (DPPH) and ferric reducing antioxidant power (FRAP), and structural modifications of *E. amoenum*, in comparison with unfermented and ultrasound-treated samples. Furthermore, pH changes and microbial growth dynamics were evaluated during the fermentation process. The findings can contribute to the development of enhanced strategies for optimizing the functionality of *E. amoenum* in food and health-related applications. Indeed, the ultrasound-assisted fermented suspension of *E. amoenum* may represent a versatile ingredient for future applications, particularly in the food, nutraceutical, cosmeceutical, and pharmaceutical sectors.

## 2. Materials and Methods

### 2.1. Materials and Reagents

Fresh flowers of *E. amoenum* were collected from the Alamut Mountains, Qazvin Province, Iran (36.45° N, 50.62° E) during the peak flowering season in May 2023. “Fresh and raw” refers to flowers processed immediately after harvesting without prior drying or storage. After cleaning, approximately 800 g of petals were air-dried at 25 ± 1 °C for 15 days under controlled laboratory conditions until constant weight was achieved.

Folin–Ciocalteu reagent, sodium carbonate, sodium nitrate, aluminum chloride, TPTZ (2,4,6-Tris(2-pyridyl)-s-triazine), ferric chloride, gallic acid, quercetin, 2,2-Diphenyl-1-picrylhydrazyl (DPPH) radical, ferrozine, ethanol, methanol, and all other reagents of analytical- or HPLC-grade were purchased from Sigma-Aldrich (St. Louis, MO, USA).

### 2.2. Preparation and Ultrasound Bath Pretreatment

The cleaned petals of *E. amoenum* were air-dried at room temperature (25 °C). Then the dried petals were well-grounded using a high-speed electric grinder and passed through a 60-mesh sieve to obtain a uniform particle size powder. The resulting powder was refrigerated in a two-layer package (aluminum and polyethylene) until further experiments. In the next step, a suspension was prepared by dispersing 6 g of *E. amoenum* powder in 100 mL of distilled water and sterilized by autoclaving at 121 °C and 15 psi for 15 min. Following sterilization and cooling, the sterile samples were subjected to an ultrasonic bath (Elmasonic S 60, Elma, Singen, Germany) operating at 37 kHz frequency and 150 W power. The ultrasound frequency of 37 kHz was selected as representative of low-intensity ultrasound conditions, previously reported to enhance mass transfer, cell permeability, and microbial metabolism while avoiding structural damage. The sonication treatment was applied for 10 min at 30 °C, with temperature control maintained using an external cooling system to prevent thermal degradation of the bioactive compounds.

### 2.3. Microorganism and Inoculum Preparation

Lyophilised *Lactobacillus acidophilus* (DSM 20079), *Lactiplantibacillus plantarum* (DSM 20179), and *Limosilactobacillus reuteri* (DSM 20016) as fermentative strains were provided by the Bioprocess Engineering Laboratory (BPEL, University of Tehran, Iran) and revived twice using sterilized MRS (Man Rogosa and Sharpe) broth (Merck, Darmstadt, Germany) at 37 °C for 16 h. All stock cultures were preserved at −20 °C in MRS broth containing 40% sterile glycerol. Following two successive transitions in sterile MRS broth, they were cultured in sterile MRS broth at 37 °C for 24 h to achieve cell concentrations of approximately 10^9^ CFU/mL.

### 2.4. Fermentation Procedure with Intermittent Ultrasonic Bath Treatment

Immediately after ultrasound pretreatment, the samples were inoculated (a total of 10^9^ CFU) with a single bacterial strain or with three different co-cultures (*L. acidophilus—L. plantarum*, *L. acidophilus*—*L. reuteri*, and *L. plantarum*—*L. reuteri*) under aseptic conditions and incubated at 37 °C for 24 h in a shaker incubator (Stuart Orbital Incubator S150). For all fermentation experiments, the total inoculation level was standardized to approximately 10^9^ CFU per fermentation system to ensure comparability between treatments. More specifically, the initial volume of the inoculum was 1% *v*/*v*; thus, in single-culture fermentations, 1% *v*/*v* (10^9^ CFU) of each bacterium was inoculated separately in every sample, but in the mixed types, co-culture (a total of 10^9^ CFU, in proportion to fifty percent of each strain) of 0.5% *v*/*v L. acidophilus* and 0.5% *v*/*v L. plantarum* or *L. reuteri* were simultaneously inoculated in a sample [24]. This strategy was adopted to maintain a constant overall microbial load across all experimental conditions and to allow meaningful comparison of fermentation performance. The inoculation level (10^9^ CFU) was determined based on viable cell counts of LAB starter cultures grown in MRS broth prior to inoculation, as quantified by plate counting on MRS agar. The viable cell count at time 0 represents the first experimentally determined plate count performed immediately after inoculation and does not correspond to the theoretical inoculum concentration.

Moreover, fermentation was conducted under mild agitation (50 rpm) to ensure homogeneous mixing of the fermentation medium, uniform nutrient availability, and consistent ultrasound energy distribution, while avoiding excessive oxygenation or shear stress. The selected LAB strains (*L. acidophilus*, *L. plantarum*, and *L. reuteri*) are aerotolerant anaerobes or facultative heterofermentative bacteria capable of maintaining metabolic activity under low-oxygen and microaerophilic conditions. The applied agitation speed was therefore selected to support stable microbial growth and metabolite production without inducing fully aerobic conditions.

During the fermentation period, ultrasonic bath treatments were applied intermittently at 4 h intervals. Each ultrasonic session lasted 2 min under the same frequency and power settings (37 kHz, 150 W) and temperature control conditions as the initial pretreatment. Samples were withdrawn immediately before each ultrasonic treatment and at the end of the fermentation period. Finally, the samples were centrifuged at 4307 *g* for 15 min at 4 °C using a benchtop refrigerated centrifuge (10410 Corporate Drive, Sugar Land, TX, USA) and then filtered. The filtrates were collected and stored at 4 °C until further analysis. All experiments were carried out in triplicate.

### 2.5. Effects of Ultrasound on the Growth of Bacterial Strains

Plate count agar using MRS agar medium was used to evaluate the effect of low-intensity ultrasound on the growth of LAB bacterial strains during the fermentation of *E. amoenum* at intervals of 4 h for 24 h. In this experiment, 100 μL of each sample was added to 9.9 mL of sterilized deionized water, and serial dilutions were made. In the next step, following the incubation period on MRS agar at 37 °C for 72 h, the logarithm of the number of colony-forming units per milliliter (log CFU/mL) was calculated. The bacterial growth curve was also plotted as log CFU/mL [25]. Samples were collected at time 0 immediately after inoculation to determine initial viable counts. To prevent the growth of undesired microorganisms, the *E. amoenum* suspension was sterilized by autoclaving prior to inoculation, and all fermentation steps were conducted under aseptic conditions. In addition, the use of selective LAB growth temperatures and the progressive acidification of the medium during fermentation further inhibited the development of competing microbial populations.

### 2.6. Total Phenolic Content (TPC) Assay

The Folin–Ciocalteu colorimetric method with a UV spectrophotometer (Lambda 25, PerkinElmer, Waltham, MA, USA) was applied to determine the TPC as described by Castangia et al. (2023) with slight modifications [26]. Briefly, 100 µL of the extract was mixed with 100 µL of 10% Folin–Ciocalteu reagent and incubated for 5 min. Then, 800 µL of 7.5% sodium carbonate solution was added. Then, the mixture was incubated in the dark at 25 °C for 30 min, and the absorbance was measured at 765 nm. Gallic acid was used as a standard, and TPC was expressed as mg of gallic acid equivalent (GAE)/g dry weight.

### 2.7. Total Flavonoid Content (TFC) Assay

The TFC was evaluated through the aluminum chloride colorimetric method, as detailed by Khosravi et al. (2024) with some modifications [27]. Briefly, 2 mL of each extract was mixed with 0.2 mL of 5% sodium nitrite and 0.2 mL of aluminum chloride. Afterward, 2 mL of 0.1 M NaOH was added to the mixture. The absorbance was recorded at 510 nm in comparison to the blank. Quercetin was utilized as the reference standard, and the TFC was reported as mg quercetin equivalents per gram of dry weight extract (mg QE/g DW).

### 2.8. Antioxidant Capacity

#### 2.8.1. DPPH Free Radical Scavenging Activity

To evaluate the antioxidant properties of the extracts, their effectiveness in scavenging DPPH radicals was measured as described by Bahrololoumi et al. (2024), with some modifications [28]. In detail, 20 µL of the extract was mixed with 1980 µL of DPPH (40 µg/mL in methanol) and incubated in the dark at room temperature for 30 min. Absorbance was then measured at 517 nm against a blank. A gallic acid calibration curve at different concentrations (0–0.010 mg/mL) was used as a reference, and antioxidant activity was expressed as mg (GAE)/g dry extract. All assays were performed in triplicate.

#### 2.8.2. Ferric-Reducing Antioxidant Power (FRAP)

The FRAP assay was conducted according to the method described by Firoznezhad et al. (2023) [2], with some modifications using a ferric complex of 2,4,6-tris(pyridin-2-yl)-1,3,5-triazine (TPTZ) and Fe^3+^. This complex was prepared by dissolving 15.62 mg of TPTZ and 16.22 mg of ferric chloride hexahydrate in 50 mL of 300 mM acetate buffer (pH 3.6). For the assay, 30 μL of the extract was added to 250 μL of the ferric complex. Following a 5 min incubation period in the dark, the absorbance was measured at 593 nm. A ferric sulfate calibration curve, ranging from 0.09 to 1.045 μM, was also prepared. The data are presented as the antioxidant capacity in mmol of ferric sulfate equivalents per gram of dry extract [2]. All measurements were performed in triplicate.

### 2.9. Fourier-Transform Infrared Spectroscopy (FT-IR) Analysis

An Avatar spectrometer (Thermo Nicolet, Waltham, MA, USA) was employed to analyze all the extracts. Prior to FT-IR analysis, fermented and ultrasound-treated and control samples were dried to eliminate residual water and obtain solid samples. FT-IR spectra were recorded on the dried samples using an ATR-FTIR spectrometer (Thermo Nicolet, Waltham, MA, USA) in the range of 4000–500 cm^−1^, with a resolution of 4 cm^−1^.

### 2.10. Statistical Analysis

All experimental assays were conducted in triplicate, and data are presented as mean ± standard deviation (SD). A two-way ANOVA followed by Tukey’s HSD post hoc test for multiple comparisons was used to compare the means between different groups. Statistical analyses were performed using Python (v 3.10). Data processing and statistical analyses were conducted using the libraries NumPy (v 1.24), pandas (v 1.5), SciPy (v 1.10), and statsmodels (v 0.14). Pearson correlation analysis was also employed to assess the relationships between bioactive compound concentrations and antioxidant activities.

## 3. Results and Discussion

### 3.1. pH Analysis During the Fermentation

**Figure 1:**
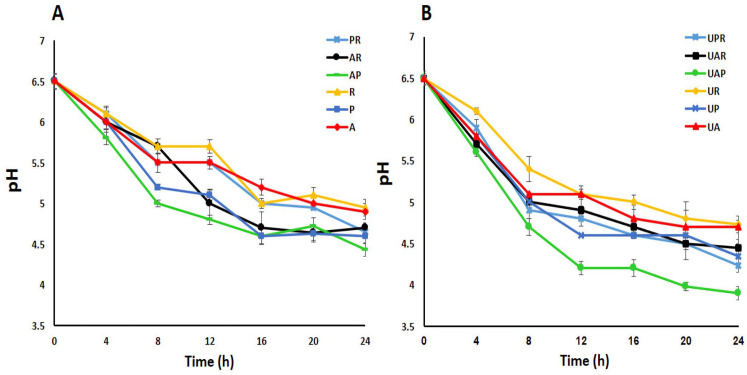
pH changes during the fermentation (**A**) and ultrasound-assisted fermentation (**B**) of *E. amoenum*. A (*L. acidophilus*); P (*L. plantarum*); R (*L. reuteri*); AP (*L. acidophilus—L. plantarum*); AR (*L. acidophilus—L. reuteri*); PR (*L. plantarum—L. reuteri*); UA (Ultrasound-assisted *L. acidophilus*); UP (Ultrasound-assisted *L. plantarum*); UR (Ultrasound-assisted *L. reuteri*); UAP (Ultrasound-assisted *L. acidophilus—L. plantarum*); UAR (Ultrasound-assisted *L. acidophilus—L. reuteri*); UPR (Ultrasound-assisted *L. plantarum—L. reuteri*).

The overall pH trend reflects the fermentative progression of *E. amoenum* in all samples and confirms the effectiveness of the low-intensity ultrasound-assisted mixed culture system (Figure 1). LAB produces lactic acid and CO_2_ during fermentation, resulting in a decrease in pH level. This pH reduction is a key indicator of successful fermentation [29], with the pH value reflecting bacterial activity and growth [24]. As illustrated in Figure 1, the pH of all fermented samples exhibited a significantly decreasing trend over the 24 h, starting from an initial pH of approximately 6.5 and dropping to values ranging between 4.95 and 3.9. The extent of pH decline varied notably among different fermentation treatments. The least pronounced pH reduction was observed in the fermented *E. amoenum* with *L. reuteri* as a single starter culture, while the most substantial pH decrease occurred in the ultrasound-assisted fermented *E. amoenum* sample through a mixed culture of *L. acidophilus* and *L. plantarum*, indicating enhanced bacterial growth and metabolic acid production, which in turn accelerate organic acid generation, driving a more rapid and extensive pH drop. Notably, in this best-performing sample, a steep and rapid pH decline was observed up to 12 h, followed by a continued but more gradual decrease from 12 to 24 h. A previous study reported that after 24 h, the pH of wheat germ fermented via a mixed culture of *L. acidophilus* and *L. plantarum* exhibited a greater reduction compared to that observed in wheat germ subjected to single LAB fermentation [24]. Utilizing mild to moderate-intensity ultrasound can enhance the efficiency of processes [30]. Meena et al. (2024) proved that low-intensity ultrasound treatment promotes the proliferation of probiotic microorganisms and increases lactic acid production [10]. Previous studies have also proved that the proliferation of LAB can be enhanced as a result of heightened activity of β-galactosidase, considering that ultrasound facilitates lactic acid yield through increased extracellular levels of β-galactosidase, which in turn raises acidity and promotes fermentation [31].

### 3.2. Effect of Low-Intensity Ultrasound on the Growth of LAB Bacterial Strains

**Figure 2:**
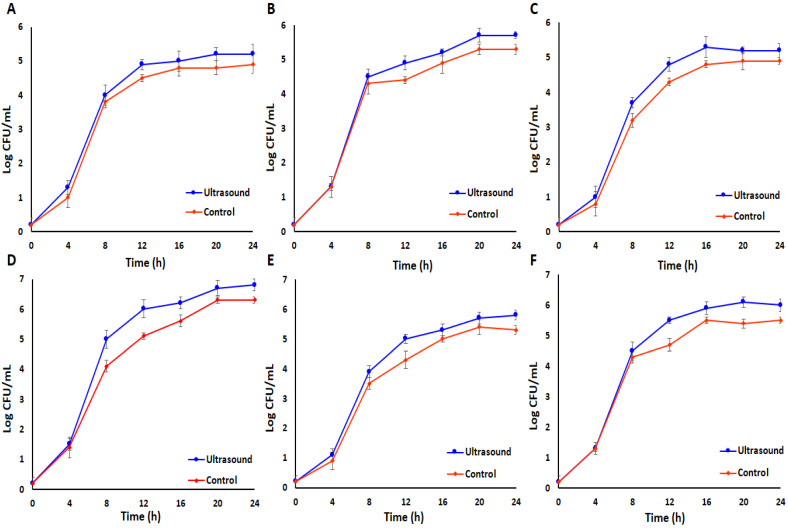
The effect of ultrasound treatment on LAB bacterial growth rate during the fermentation of *E. amoenum* at 37 °C for 24 h in single (*L. acidophilus* (**A**), *L. plantarum* (**B**)*, L. reuteri* (**C**)), and mixed (*L. acidophilus*—*L. plantarum* (**D**), *L. acidophilus*—*L. reuteri* (**E**), and *L. plantarum*—*L. reuteri* (**F**)) cultures, compared to fermented control samples without ultrasound exposure.

An investigation into the effect of low-intensity ultrasound on the growth of single and co-cultured LAB strains during the fermentation process (24 h) of *E. amoenum* was conducted by evaluating changes in log CFU/mL. Initially, the increase was gradual during the incubation phase (0–4 h), followed by a marked rise during the logarithmic phase (4–12 h), leading up to the stationary phase. Furthermore, the log CFU/mL of ultrasound-treated samples was significantly elevated compared to control samples after the logarithmic phase (*p* < 0.05), indicating that low-intensity ultrasound facilitates fermentation by LAB (Figure 2).

Previous research indicated that ultrasound treatment significantly affects fermentation kinetics [10,32]. Cavitation induced by high-intensity ultrasound waves can create elevated temperatures and pressures in liquids, leading to direct damage to bacterial cell walls. It can also enhance the decomposition of water molecules, produce free radicals, and impair DNA and enzymes in bacteria [33]. Furthermore, the antibacterial effects of ultrasound are significantly influenced by its intensity, which means that, at suitable ultrasonic intensities, bacterial metabolism and growth can be stimulated. For example, the application of ultrasound at 100 W, 30 kHz, and 25% amplitude for 15 min has been shown to promote the growth and replication of *L. plantarum* AF1 during the fermentation of milk, attributed to improved permeability of the cell membrane and the enhanced antioxidant properties of the bacterium [34]. Another study indicated that intermittent low-intensity ultrasound treatment (0.167 W/cm^2^) combined with *Lacticaseibacillus paracasei* for 10 min at 8 h intervals promotes bacterial growth, resulting in a 16.72% enhancement in decalcification and a 33.45% increase in deproteinization, while also reducing the fermentation duration of crab shells to 48 h [31]. Under low-intensity ultrasound treatment, the growth of bacterial strains was observed to increase rather than be inhibited. This effect may be attributed to several mechanisms, including the fact that low-intensity ultrasound can improve the permeability of cell membranes and facilitate mass transfer, thereby promoting microbial growth and process efficiency [10,35]. Furthermore, it can improve the transport of oxygen and essential nutrients for microbial growth [36] and also induces stable cavitation, causing repairable damage to bacterial cells, which may alter the bacterial state and accelerate proliferation [37]. Additionally, ultrasound treatment significantly enhanced bacterial growth in co-culture systems beyond that observed in monocultures, suggesting that ultrasound exposure intensifies synergistic interactions among bacterial strains, possibly by modulating cellular stress responses and metabolic cooperation. Although mixed-culture fermentations demonstrated enhanced bioactive compound release and antioxidant capacity compared to monocultures, strain-specific growth dynamics were not individually monitored in the present study. Therefore, the observed synergistic effects reflect the collective metabolic activity of the co-cultures rather than the contribution of individual strains. Future investigations employing strain-specific enumeration methods, such as selective media, qPCR, or metagenomic approaches, would be valuable to further elucidate interspecies interactions and microbial succession dynamics during ultrasound-assisted co-culture fermentation. Consistent with our findings, previous studies have reported that low-intensity ultrasound can stimulate the growth of LAB during fermentation. For example, Chen et al. reported that after 24 h of fermentation of ginkgo kernel juice, the viable cell counts of *L. plantarum* Y2 increased by 5.06%, 5.05%, and 2.19% under ultrasound power densities of 173.88, 115.50, and 84.42 W/L, respectively, compared to non-sonicated controls. This study, although conducted on a different substrate and bacterial strain, supports the general role of low-intensity ultrasound in enhancing LAB proliferation under controlled conditions [30]. Ultrasound treatment exerts a beneficial effect on the fermentation efficiency of microorganisms by stimulating cellular growth and enhancing enzyme activity and metabolism, thereby enhancing the overall fermentation process [8,9]. A 5 min ultrasound (35 kHz) before the fermentation of black carrot juice led to a reduction in the number of LAB, while 15- and 30 min pretreatment led to an enhancement [38]. Controlled ultrasound treatments (23 kHz, 10 µm amplitude for 3 and 5 min) induced a maximum 1.09 log CFU/mL elevation in *Lactobacillus brevis* (LMG 6906) cell counts, a 58.17–82.59% enhancement in specific growth rate, and a 16–36.25% augmentation in cell membrane permeability, which are correlated with a 0.51 decrease in pH level [39]. In addition, low-intensity ultrasound was observed to enhance growth parameters, including specific growth rate and logarithmic phase duration, as well as lactic acid production, cellular reproduction, and substrate consumption in *Lactobacillus casei* [40]. Overall, ultrasound can effectively speed up the fermentation process, decrease fermentation time, improve microorganism growth, and enhance biomass yields [41].

### 3.3. Total Polyphenol Content (TPC) and Total Flavonoid Content (TFC)

TPC and TFC were quantitatively assessed throughout the ultrasound treatment and LAB fermentation process of *E. amoenum* at 4, 8, 12, 16, 20, and 24 h under four distinct conditions, including single-culture fermentation, co-culture fermentation, and their respective treatments combined with low-intensity ultrasound. The detailed data for TPC and TFC are presented separately in Table 1 and Table 2, respectively. Across all fermentation modes, there was a statistically significant increase in both TPC and TFC levels compared to unfermented controls and ultrasound-treated samples (*p* < 0.05), with the ultrasound-assisted co-cultured LAB fermentation exhibiting the highest accumulation.

During LAB fermentation, the increase in measurable phenolic content primarily reflects the release of phenolic compounds bound to the plant cell wall matrix. Throughout this process, microorganisms utilize enzymes such as cellulases and pectinases to break down cell walls, facilitating the release of phenolics. In *E. amoenum*, a significant fraction of phenolics is associated with polysaccharides and structural components of the cell wall through ester or glycosidic linkages. Fermentation-induced acidification, together with enzyme-mediated hydrolysis involving esterases, glycosidases, and phenolic acid–modifying enzymes, promotes matrix loosening and facilitates the liberation of these bound phenolics into the soluble fraction [7]. Specifically, Lactobacillaceae, as key organisms in food fermentation, metabolize polyphenolic compounds via reductases, decarboxylases, and glycosidases [42]. In addition, microorganisms possess the capability to synthesize flavonoids from carbohydrates and amino acids via enzymatic processes. The synergistic action within the mixed bacterial culture likely enhances enzymatic hydrolysis of *E. amoenum* cell walls, facilitating greater phenolic release and enhancing metabolic biosynthesis. Additionally, ultrasound improves the permeability of cell membranes and facilitates mass transfer, thereby accelerating extraction kinetics and microbial metabolism [30]. This resulted not only in elevated total phenolic and flavonoid yields but also in a significantly reduced extraction time relative to controls [9]. To comprehensively compare the total phenolic and flavonoid contents, a time-course analysis was conducted at identical time points for the different fermented samples, the ultrasound-treated sample, and the untreated control, with particular emphasis on the 12 h time point, corresponding to the peak bioactive response in ultrasound-assisted co-culture fermentation. In the ultrasound-treated sample, a significant increase in TPC and TFC was observed within the first 4 h; thereafter, the rate of increase showed a declining trend, eventually reaching a value of around 160 mg GAE/g of dry weight and 58 mg QE/g of dry weight, respectively, after 24 h. The initial significant increase within the first 4 h is attributed to the immediate release of soluble phenolic and flavonoid compounds. The subsequent decline in the rate of increase is likely due to the exhaustion of readily extractable compounds, potential degradation of sensitive phenolic and flavonoid compounds under prolonged exposure to sonication, and the establishment of an equilibrium between extraction and decomposition. The final value of both TPC and TFC after 24 h suggests the maximum achievable extraction yield under the given ultrasonic conditions. Peak concentrations of TPC and TFC were consistently observed at 12 h in the ultrasound-assisted mixed fermentation using *L. acidophilus* and *L. plantarum*, reaching approximately 299 mg GAE/g of dry weight and 88 mg QE/g of dry weight, respectively. Following this peak, a gradual decline in TPC was observed, likely due to acidification-induced degradation and polymerization of phenolics, as well as possible microbial catabolism under the fermented acidic environment. Nevertheless, TPC and TFC levels remained significantly elevated relative to controls and ultrasound-treated samples throughout the fermentation period, underscoring the effectiveness of this integrated bioprocessing strategy.

Similar findings have been reported in recent studies demonstrating increased phenolic and flavonoid content in plant materials following fermentation and ultrasound-assisted fermentation. The co-fermentation process involving *L. paracasei* ZH8 and *Lactococcus lactis* subsp. *lactis* YM313 in wolfberry–longan juice markedly enhanced the total levels of phenolics and flavonoids, thereby improving the antioxidant properties [19]. Considering the limited bioavailability of plant phenolic compounds, with only 5–10% being effectively digested [40], microbial enzymes could release conjugated phenolics from plant cell walls, thus improving their bioavailability [7]. Optimization of microbial strains and fermentation parameters can markedly elevate bioactive phenolic yields in fermented products [42,43]. Ultrasound-assisted fermentation of *Citrus reticulata* peel powder significantly increased total protein and carotenoid levels by 85.26% and 179.68%, respectively, compared to non-fermented *Citrus reticulata* peel powder, and outperformed conventionally fermented samples. Differential metabolite analysis identified 521 compounds, primarily organic acids, lipids, and flavonoids [44]. Similarly, ultrasonicated-assisted rice lees fermented with *Lactobacillus helveticus* (URLH-48) showed the highest TPC (112.1 mg GAE/mL) in comparison with control and other treatments [45]. The integration of fermentation pre-treatment with ultrasound-assisted extraction notably enhanced the recovery of TPC from mango leaves (ranging from a 20% to 52% increase), resulting in improved antioxidant and antidiabetic properties [43]. Furthermore, sonication pretreatments considerably enhanced the transformation of free phenolic acids obtained from pineapple peel fiber throughout the fermentation process by *Lactobacillus bulgaricus* and *Streptococcus thermophilus*, especially boosting the levels of ferulic acid, caffeic acid, and 5-hydroxyflavone. This underscores the beneficial impact of sonication-assisted fermentation in facilitating the hydrolysis of conjugated phenolics into their free forms [46]. Moreover, in contrast to the non-sonicated sauce, the ultrasound-assisted (low-ultrasonic power densities) fermentation of *Porphyra yezoensis* sauce with *L. plantarum* demonstrated an increase of about 58% in TPC and 27% in TFC, reaching 92.38 mg GEA/g of dry weight and 111.08 mg RE/g of dry weight, respectively [47]. In comparison to the control group, low-intensity ultrasound treatment significantly enhanced (*p* < 0.05) TFC (30.27% and 12.00% increase) during mycelium fermentation [48]. In addition, findings from another study demonstrated that the incorporation of ultrasound-assisted fermentation led to a significant increase in the TPC, TFC, and antioxidant activity of grape juice compared to both fermented and non-fermented grape juice (*p* < 0.001) [17].

### 3.4. Antioxidant Potential

The antioxidant capacity of *E. amoenum* samples was evaluated using DPPH and FRAP assays at multiple fermentation time points. Both assays showed a significant enhancement in antioxidant activity for all treatments compared to the unfermented controls. Among them, the highest activity was observed for the ultrasound-assisted co-cultured fermentation involving *L. acidophilus* and *L. plantarum*, followed by fermentation with *L. plantarum* and *L. reuteri*, and then by those with *L. acidophilus* and *L. reuteri*. This enhancement can be ascribed to both quantitative and qualitative improvements, including increased soluble antioxidant phenolics concentrations and potential microbial biotransformation into metabolites with stronger radical scavenging capacity. During the fermentation process, LAB consume nutrients from the substrate to promote their growth, while also enabling the bioconversion of compounds through structural alterations, which in turn enhances the levels of metabolites with significant antioxidant properties [49]. Furthermore, prior research indicates that specific metabolites generated by starter strains during fermentation may significantly enhance antioxidant activity. For example, bioactive compounds produced during fermentation, such as peptides and xylooligosaccharides [41], and also antioxidant enzymes such as superoxide dismutase and catalase, can play a critical role in strengthening the antioxidant capacity [7]. In addition, pretreatment and intermittent exposure to ultrasound improve extraction efficacy and promote microbial uptake and metabolic transformation of phenolics, thereby optimizing the antioxidant profile.

**Figure 3:**
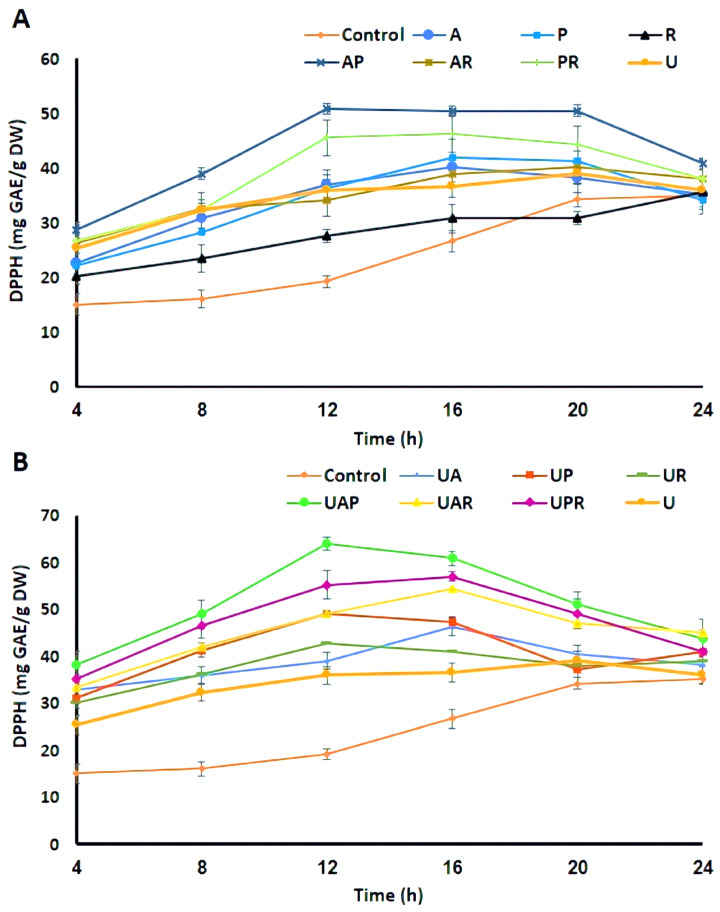
DPPH free radical scavenging activity of the (**A**) unfermented control, ultrasound-treated sample (U), and fermented *E. amoenum* using (A (*L. acidophilus*); P (*L. plantarum*); R (*L. reuteri*); AP (*L. acidophilus—L. plantarum*); AR (*L. acidophilus—L. reuteri*); PR (*L. plantarum—L. reuteri*)); (**B**) unfermented control, ultrasound-treated sample (U), and ultrasound-assisted fermentated *E. amoenum* (UA (Ultrasound-assisted *L. acidophilus*); UP (Ultrasound-assisted *L. plantarum*); UR (Ultrasound-assisted *L. reuteri*); UAP (Ultrasound-assisted *L. acidophilus—L. plantarum*); UAR (Ultrasound-assisted *L. acidophilus—L. reuteri*); UPR (Ultrasound-assisted *L. plantarum—L. reuteri*)).

**Figure 4:**
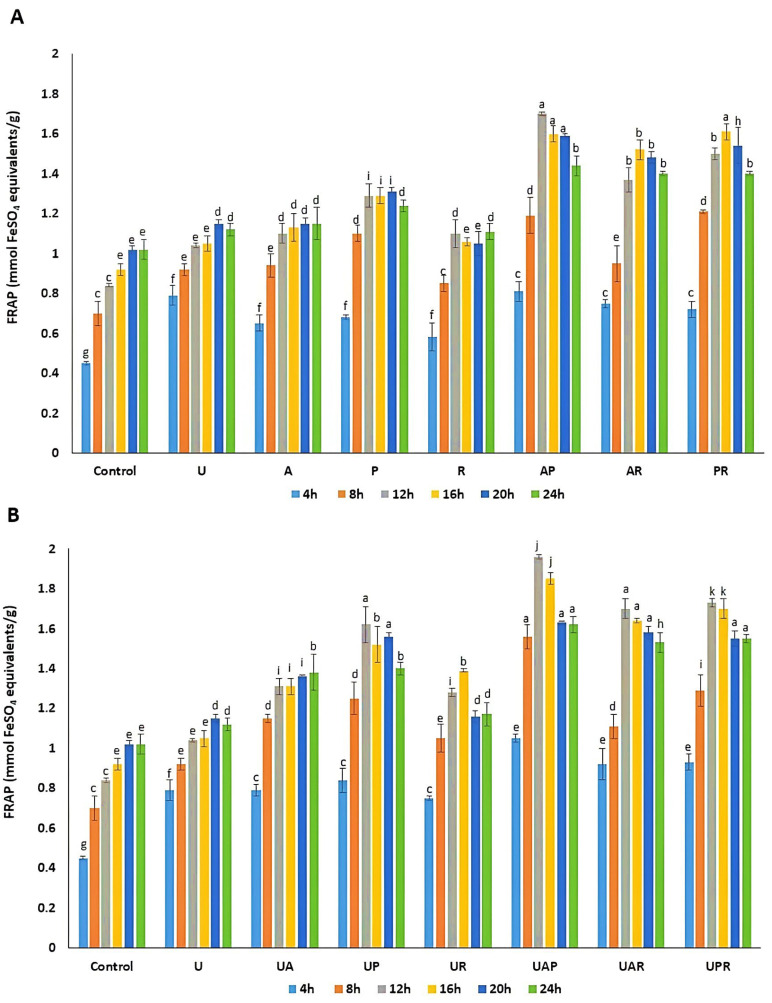
FRAP analysis of the (**A**) unfermented control, ultrasound-treated sample (U), and fermented *E. amoenum* using (A (*L. acidophilus*); P (*L. plantarum*); R (*L. reuteri*); AP (*L. acidophilus—L. plantarum*); AR (*L. acidophilus—L. reuteri*); PR (*L. plantarum—L. reuteri*)); (**B**) unfermented control, ultrasound-treated sample (U), and ultrasound-assisted fermentated *E. amoenum* (UA (Ultrasound-assisted *L. acidophilus*); UP (Ultrasound-assisted *L. plantarum*); UR (Ultrasound-assisted *L. reuteri*); UAP (Ultrasound-assisted *L. acidophilus—L. plantarum*); UAR (Ultrasound-assisted *L. acidophilus—L. reuteri*); UPR (Ultrasound-assisted *L. plantarum—L. reuteri*)). Mean values  ±  standard deviations are reported (*n* = 3). In each graph, the same letter indicates not statistically different values (*p* > 0.05 among values with same letter and *p* < 0.05 versus values with different letters).

DPPH radical scavenging activity peaked at approximately 64.02 ± 1.67 mg GAE/g of dry weight after a 12 h fermentation period, compared to the peak of the unfermented control (35.08 ± 1.56 mg GAE/g of dry weight) at 24 h and the peak of the ultrasound-treated sample (39.10 ± 2.32 mg GAE/g of dry weight) at 20 h. Similarly, FRAP values in the fermented samples reached around 1.96 mmol FeSO_4_ equivalents/g of dry weight at 12 h, while the control peaked later at roughly 1.02 mmol FeSO_4_ equivalents/g of dry weight at 24 h, and the ultrasound-treated *E. amoenum* showed the peak value of 1.15 mmol FeSO_4_ equivalents/g of dry weight at 20 h of the treatment. These data are presented in Figure 3 and Figure 4, respectively. Following the peak at 12 h, a moderate decline in antioxidant activity was observed in some samples, likely due to partial degradation of phenolic compounds under acidic and oxidative stress caused by extended fermentation. Nonetheless, antioxidant activity remained substantially elevated relative to unfermented samples throughout the fermentation period, supporting the efficiency of ultrasound-assisted co-cultured LAB fermentation in maximizing DPPH antioxidant potential of *E. amoenum*. The antiradical properties of fermented foods are influenced by several factors, including fermentation duration, pH levels, microbial strains, dissolved oxygen concentration, temperature, and others [41]. Recent findings indicate that lactic acid fermentation-assisted extraction improves the recovery of antioxidants from fig leaves compared to single-solvent extraction methods applied to non-fermented samples [50]. Consistent with prior findings, LAB co-culture systems significantly enhance the antioxidant activity of plant extracts [24,27]. This improvement is correlated with elevated phenolic content, primarily resulting from the enhanced capacity of co-culture processes to transform bound and conjugated polyphenols into their free form, relative to single-culture methodologies [7,27,51]. Consequently, this process leads to an increased number of hydroxyl groups, which are considered key contributors to the observed beneficial activity [27]. Further findings regarding the enhancement of antioxidant activity in wheat germ through mixed LAB fermentation compared to single fermentation revealed a significant rise in DPPH antioxidant activity, increasing from 51.18% in raw wheat germ to 89.76% in mixed fermented wheat germ using *L. plantarum* and *L. acidophilus* [24]. Furthermore, co-fermentation of rambutan juice using *L. plantarum* and *Limosilactobacillus fermentum* improved both antioxidant activity and flavor complexity [52]. Additionally, ultrasound-assisted solid-state fermentation of *citrus reticulata* peels using *Aspergillus niger* maximized TPC, TFC, ABTS, and DPPH radical scavenging activity by 29.6%, 31.2%, 48.2%, and 71.8%, respectively, in contrast to the non-fermented sample [53]. In addition, the in vitro antioxidant activity of fermented Ginkgo kernel juice with *L. plantarum* was enhanced through ultrasonication, which facilitated the metabolism of phenolic acids, including ferulic acid, chlorogenic acid, and caffeic acid [30]. In another study by Ruan et al. (2020), ultrasound-assisted liquid-state fermentation by *Bacillus subtilis* enhanced the in vitro antioxidant capacity of soybean meal [54]. Valorization of corn processing by-products through ultrasound-assisted fermentation led to enhanced levels of TPC and TFC, thereby increasing antioxidant activity [21]. Another investigation on the evaluation criteria for various fermentation stages under low-power ultrasonication indicated that the *P. yezoensis* sauce produced a higher quantity of phenolic compounds and demonstrated enhanced antioxidant properties in the sonicated sample during the logarithmic phase of *L. plantarum* [47]. This comprehensive analysis confirms that ultrasound-assisted mixed culture LAB fermentation improves the extraction and antioxidant potential of *E. amoenum* phenolics and flavonoids. The synergistic action among LAB bacterial strains, especially *L. acidophilus* and *L. plantarum*, enhances compound release and bioactivity, while ultrasound treatment positively influences cell wall disruption and microbial metabolism.

### 3.5. Correlations

**Figure 5:**
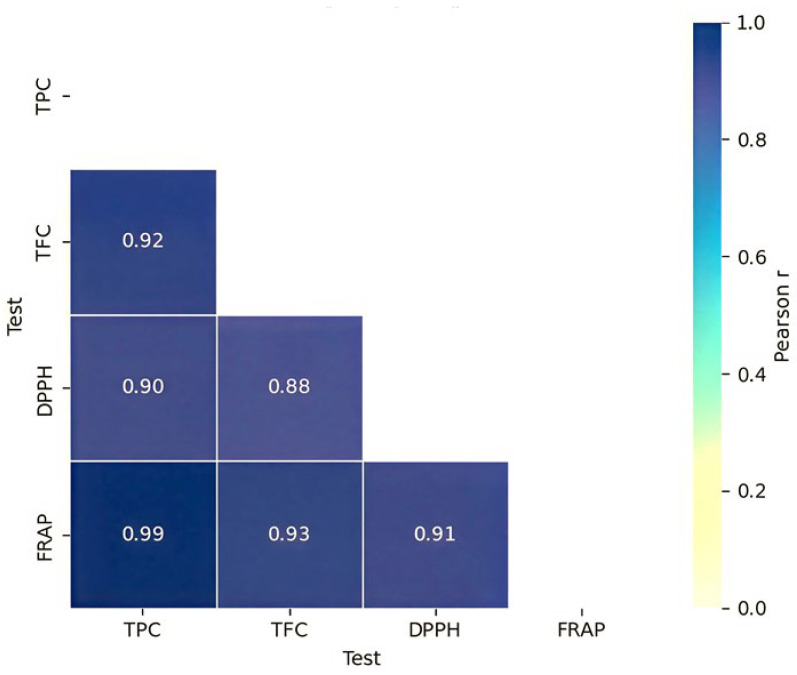
Correlation between TPC, TFC, and antioxidant activities (DPPH and FRAP).

The Pearson correlation assay was applied to analyze the correlation between the phenolic and flavonoid contents and antioxidant activities (DPPH and FRAP) of different fermented extracts (Figure 5). Strong positive correlations were observed between TPC and TFC and both DPPH radical scavenging activity (r = 0.90 and r = 0.88, respectively) and FRAP (r = 0.99 and r = 0.93, respectively). These results align with Khosravi et al. (2024), who correlated total polyphenol and flavonoid with DPPH and the metal chelating ability of fermented date by-product [27]. Similarly, Akbari et al. (2023) revealed a strong correlation between TPC and TFC with DPPH and ABTS scavenging activity of fermented ethanolic extract of corn bran samples using *L. plantarum* and *L. reuteri* [55]. Additionally, Carmo Brito et al. (2017) found a strong positive correlation between total phenolic compounds and total anthocyanins with the ABTS scavenging activity in fermented cocoa beans [56].

### 3.6. FT-IR

**Figure 6:**
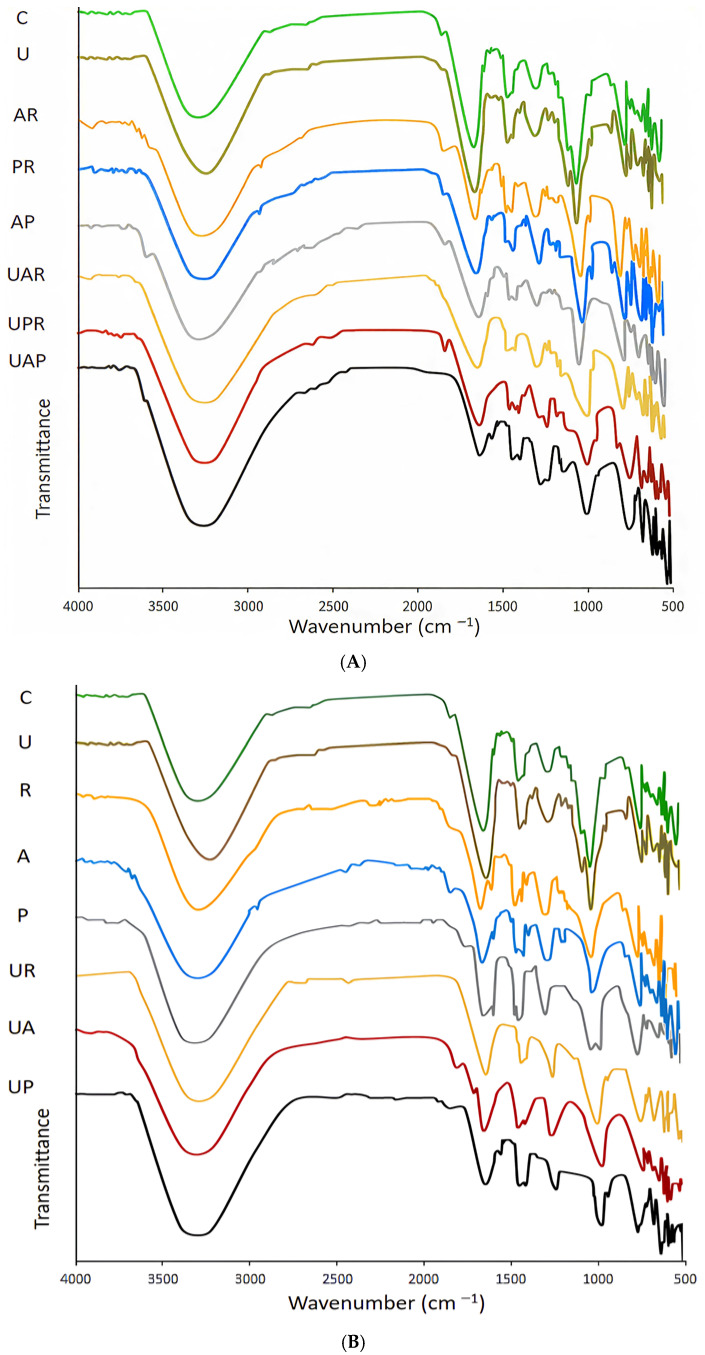
(**A**) FTIR spectra of the unfermented control *E. amoenum* sample (C), the ultrasound-treated sample (U), the mixed culture fermented samples (*L. acidophilus*—*L. reuteri* (AR), *L. plantarum—L. reuteri* (PR), and *L. acidophilus*—*L. plantarum* (AP)), and the ultrasound-assisted mixed culture fermented samples (*L. acidophilus*—*L. reuteri* (UAR), *L. plantarum—L. reuteri* (UPR), and *L. acidophilus*—*L. plantarum* (UAP)). (**B**) Spectra of the control unfermented *E. amoenum* sample (C); the ultrasound-treated sample (U); the single culture fermented samples (*L. acidophilus* (A), *L. reuteri* (R), and *L. plantarum*) (P); and the ultrasound-assisted single culture fermented samples (*L. acidophilus* (UA), *L. reuteri* (UR), and *L. plantarum* (UP)).

The variations in the chemical bonds and functional groups of ultrasound-treated, fermented, and ultrasound-assisted fermented *E. amoenum* samples are presented in Figure 6. FTIR spectroscopy provides a valuable method for examining bacterial responses to ultrasound treatment [39]. As shown in Figure 6A and Figure 6B, ultrasound-assisted fermentation induced more pronounced spectral shifts compared to ultrasound-treated and fermentation alone, particularly in regions associated with hydroxyl and carbonyl functional groups. Specifically, the control *E. amoenum* sample exhibits a strong, broad absorption band in the 3200–3400 cm^−1^ region, associated with O–H stretching vibrations in hydroxyl-rich compounds such as polysaccharides and polyphenols [55,57]. Furthermore, distinct bands in the 1700–1600 cm^−1^ region stem from C=O stretching and aromatic C=C bonds in polyphenols and lignin-derived structures [27]. In addition, bands observed around 1200–1000 cm^−1^ are characteristic of C–O and C–C stretching in carbohydrates, highlighting the integrity of the cell wall polysaccharide network. As can be seen in the figure, the ultrasound-treated *E. amoenum* sample exhibits a distinct increase in intensity and slight band broadening in the 3200–3400 cm^−1^ region. This indicates enhanced release of hydroxyl-containing compounds such as polyphenols from within the cells. The ultrasound-induced cavitation creates temporary pores and increases cell-wall permeability, allowing these compounds to be more efficiently extracted without generating new hydroxyl groups through chemical reactions. Moving to the 1600–1700 cm^−1^ range, the intensity remains largely unchanged or shows a slight increase. This confirms that under mild sonication, the covalent bonds of conjugated carbonyls and aromatic rings remain intact. Ultrasound facilitates their physical release but does not degrade their core chemical structures. In the polysaccharide sensitive region of 1000–1200 cm^−1^, no significant change in peak shape, position, or overall intensity is noted. Mild ultrasound causes physical disaggregation, such as the loosening of the cell-wall network and partial breakdown of weak intermolecular interactions, but does not substantially hydrolyze the covalent glycosidic bonds of cellulose, hemicellulose, or pectin. Consequently, the polysaccharide fingerprint in the infrared spectrum remains similar to that of the untreated control. Furthermore, from the figure, it is evident that fermentation induces significant biochemical and structural changes, which are prominently observed as increased intensity and shifts in O–H bands, reflecting the enhanced level of free phenolic acids resulting from the activity of the enzymes produced by the LAB during fermentation and modification of phenolic compounds [55]. Microbial enzymes such as cellulases and pectinases disrupt the plant matrix, enhancing the extractability of antioxidants and increasing the phenolic content. The effectiveness of antioxidants is determined by structural characteristics, including the quantity and arrangement of hydroxyl groups on the ring structures, and the degree of unpaired electron delocalization within the oxidized phenolic intermediate [58]. Prior research indicates that phenolic compounds were primarily hydroxylated during the fermentation process using LAB [49]. Furthermore, the unfermented control spectrum exhibits a notably higher peak intensity in the 1600–1700 cm^−1^ region, which corresponds primarily to C=O stretching vibrations of phenolic acids, esters, and other carbonyl-containing compounds, as well as aromatic C=C stretching vibrations commonly found in lignin and polyphenols. The elevated intensity in the unfermented sample indicates a greater abundance of intact carbonyl and aromatic structures. Fermentation results in a marked decrease in peak height in this region, indicating enzymatic degradation and biochemical transformation of these carbonyl and aromatic compounds. Specifically, microbial activity during fermentation hydrolyzes carbonyl-containing esters and modifies phenolic structures, resulting in reduced intensities of these functional groups. A parallel trend is also observed in the polysaccharide region near 1000 cm^−1^, where the peak intensity is similarly diminished in the fermented samples compared to the control. This band is attributed to C–O stretching vibrations in cellulose, hemicellulose, and other structural polysaccharides. The reduction in peak height further reflects microbial enzymatic depolymerization of the polysaccharide matrix, promoting the breakdown of the plant cell wall through cleaving the intramolecular glycosidic bonds (β-1,4) and facilitating the release of bound phenolic antioxidants [55]. The observed effects are significantly more pronounced in mixed bacterial cultures, likely attributable to their synergistic interactions, which enhance metabolic activity and promote superior growth compared to monocultures under the same conditions. Ultrasound-assisted fermented samples exhibited the most pronounced FTIR spectral shifts. These alterations confirm extensive depolymerization of polysaccharides and maximal release of bioactive phenolic compounds and are directly correlated with the experimentally observed enhancement in the antioxidant activity and total phenolic content of fermented samples.

## 4. Conclusions

This study elucidated the significant impact of low-intensity ultrasound-assisted mixed LAB fermentation on the microbial dynamics and bioactive profile of *E. amoenum* during a 24 h fermentation period. Ultrasound-assisted co-culture LAB fermentation procedures, especially the one combining *L. acidophilus* and *L. plantarum*, not only enhanced bacterial proliferation and metabolite biosynthesis, reflected by a pH drop and increased CFU/mL counts, but also significantly elevated total phenolic and flavonoid contents, as well as DPPH and FRAP antioxidant capacities, compared to ultrasound-treated samples and unfermented controls. Importantly, the ultrasound treatment accelerated the attainment of peak bioactive levels, achieving in 12 h what control samples could not reach even after 24 h, highlighting the superior efficiency of the ultrasound-assisted mixed LAB fermentation in enhancing both the quantity and rate of bioactive compound extraction. Furthermore, FTIR analysis confirmed structural modifications and alterations in polyphenolic functional groups, underpinning the enhanced release of bioactive compounds. Overall, the synergistic effects of low-intensity ultrasound treatment and co-cultured LAB fermentation present an efficient bioprocessing technology for maximizing bioactive metabolites and functional properties of *E. amoenum*. In particular, owing to its enhanced antioxidant power and phenolic content, it could be incorporated into functional foods and beverages, developed as a nutraceutical extract, or employed in cosmetic and dermatological formulations aimed at oxidative stress mitigation. Furthermore, its bioactive-rich profile may support pharmaceutical applications following appropriate downstream processing and safety evaluation.

## Figures and Tables

**Table 1:** Total polyphenol content (TPC) of unfermented, ultrasound-treated, fermented, and ultrasound-assisted fermented *E. amoenum* extracts. Data are expressed as mean ± SD. For each group, means with different lowercase letters differ significantly (*p* < 0.05).

Sample	TPC (mg GAE/g DW)					
	Time (4 h)	Time (8 h)	Time (12 h)	Time (16 h)	Time (20 h)	Time (24 h)
Control	67.91 ^m^ ± 2.23	109.12 ^h^ ± 1.61	121.33 ^d^ ± 0.55	133.91 ^i^ ± 3.14	145.77 ^l^ ± 0.44	147.23 ^l^ ± 0.17
Ultrasound	105.89 ^h^ ± 0.78	134.31 ^i^ ± 2.31	149.88 ^l^ ± 2.91	150.16 ^s^ ± 1.43	158.55 ^j^ ± 0.64	160.28 ^e^ ± 1.12
Single fermentation						
A	93.32 ^k^ ± 1.33	136.68 ^i^ ± 2.44	157.56 ^j^ ± 0.66	163.79 ^e^ ± 2.06	163.31 ^e^ ± 1.15	164.93 ^e^ ± 0.51
P	98.27 ^k^ ± 2.69	163.12 ^e^ ± 1.57	179.18 ^p^ ± 0.34	183.32 ^p^ ± 1.78	188.15 ^q^ ± 3.88	177.51 ^r^ ± 3.31
R	87.27 ^t^ ± 0.17	121.22 ^d^ ± 2.09	147.21 ^l^ ± 2.91	151.16 ^s^ ± 0.63	149.66 ^l^ ± 3.03	162.79 ^e^ ± 0.74
Mixed fermentation						
AP	123.43 ^d^ ± 3.21	167.19 ^e^ ± 3.99	241.76 ^a^ ± 2.58	251.66 ^b^ ± 0.15	240.23 ^a^ ± 3.81	221.39 ^c^ ± 0.37
AR	111.22 ^h^ ± 0.72	133.612 ^i^ ± 3.07	197.21 ^f^ ± 0.47	218.11 ^c^ ± 3.12	211.69 ^g^ ± 2.01	201.11 ^f^ ± 3.02
PR	106.11 ^h^ ± 2.29	181.88 ^p^ ± 1.36	208.61 ^g^ ± 2.03	225.71 ^n^ ± 1.93	221.33 ^c^ ± 0.38	235.33 ^o^ ± 3.12
Ultrasound-assisted single fermentation						
UA	116.99 ^w^ ± 1.08	179.21 ^p^ ± 0.17	192.61 ^q^ ± 0.31	188.30 ^q^ ± 0.21	198.73 ^f^ ± 3.91	197.43 ^f^ ± 0.88
UP	123.04 ^d^ ± 2.77	189.33 ^q^ ± 3.71	231.12 ^o^ ± 0.11	217.35 ^x^ ± 0.34	223.31 ^c^ ± 1.25	201.33 ^f^ ± 3.12
UR	111.44 ^h^ ± 1.52	151.94 ^s^ ± 2.82	173.87 ^r^ ± 1.33	199.11 ^f^ ± 2.16	165.21 ^e^ ± 1.53	161.33 ^j^ ± 0.72
Ultrasound-assisted mixed fermentation						
UAP	156.31 ^j^ ± 2.17	218.55 ^c^ ± 0.39	299.42 ^u^ ± 0.89	278.27 ^v^ ± 0.61	233.48 ^o^ ± 2.33	231.87 ^o^ ± 1.69
UAR	137.31 ^i^ ± 0.16	159.56 ^j^ ± 1.07	241.91 ^a^ ± 2.03	249.66 ^b^ ± 1.77	226.15 ^n^ ± 1.91	219.11 ^c^ ± 0.16
UPR	135.78 ^i^ ± 3.07	185.11 ^y^ ± 0.57	248.21 ^b^ ± 1.19	223.88 ^c^ ± 0.39	220.81 ^c^ ± 1.77	215.33 ^x^ ± 3.12

Single culture fermentation (*L. acidophilus* (A), *L. plantarum* (P), *L. reuteri* (R)); mixed culture fermentation ((*L. acidophilus*—*L. plantarum* (AP), *L. acidophilus*—*L. reuteri* (AR), and *L. plantarum*—*L. reuteri* (PR)); ultrasound-assisted single fermentation using *L. acidophilus* (UA), *L. plantarum* (UP), *L. reuteri* (UR); ultrasound-assisted mixed fermentation using *L. acidophilus*—*L. plantarum* (UAP), *L. acidophilus*—*L. reuteri* (UAR), and *L. plantarum*—*L. reuteri* (UPR).

**Table 2:** Total flavonoid content (TFC) of unfermented, ultrasound-treated, fermented, and ultrasound-assisted fermented *E. amoenum* extracts. Data are expressed as mean ± SD. For each group, means with different lowercase letters differ significantly (*p* < 0.05).

Sample	TFC (mg QE/g DW)					
	Time (4 h)	Time (8 h)	Time (12 h)	Time (16 h)	Time (20 h)	Time (24 h)
Control	20.12 ^g^ ± 1.33	32.44 ^h^ ± 0.06	42.11 ^c^ ± 0.41	50.12 ^e^ ± 1.11	50.87 ^e^ ± 1.66	53.11 ^e^ ± 0.41
Ultrasound	34.16 ^h^ ± 2.03	43.28 ^c^ ± 0.91	46.59 ^k^ ± 1.17	50.08 ^e^ ± 1.84	55.77 ^d^ ± 2.84	58.61 ^d^ ± 0.31
Single fermentation						
A	39.91 ^c^ ± 1.25	41.54 ^c^ ± 3.01	48.33 ^e^ ± 1.29	52.22 ^e^ ± 2.83	50.51 ^e^ ± 0.41	60.32 ^d^ ± 1.31
P	40.24 ^c^ ± 0.14	55.91 ^d^ ± 0.94	54.91 ^i^ ± 2.03	60.91 ^d^ ± 1.82	64.91 ^f^ ± 2.41	65.97 ^f^ ± 0.58
R	39.38 ^c^ ± 1.09	40.51 ^c^ ± 3.65	42.96 ^c^ ± 3.61	51.55 ^e^ ± 0.64	59.81 ^d^ ± 0.23	61.89 ^f^ ± 2.93
Mixed fermentation						
AP	43.42 ^c^ ± 3.25	58.53 ^d^ ± 0.15	69.57 ^a^ ± 3.68	75.87 ^b^ ± 0.77	72.13 ^a^ ± 0.97	74.72 ^a^ ± 1.95
AR	42.18 ^c^ ± 1.59	49.91 ^e^ ± 1.25	52.91 ^e^ ± 1.25	56.91 ^d^ ± 1.25	69.21 ^a^ ± 0.67	66.91 ^f^ ± 4.05
PR	44.53 ^c^ ± 3.11	55.11 ^d^ ± 4.08	70.99 ^a^ ± 3.64	70.21 ^a^ ± 2.45	69.44 ^a^ ± 3.09	70.1 ^a^ ± 0.19
Ultrasound-assisted single fermentation						
UA	45.88 ^k^ ± 2.55	50.22 ^e^ ± 0.55	51.21 ^e^ ± 1.61	63.32 ^f^ ± 0.63	66.87 ^f^ ± 1.39	63.25 ^f^ ± 3.04
UP	46.61 ^k^ ± 1.99	60.32 ^d^ ± 3.15	69.12 ^a^ ± 0.16	70.23 ^a^ ± 3.31	69.87 ^a^ ± 1.39	65.32 ^f^ ± 0.15
UR	45.82 ^k^ ± 0.78	50.191 ^e^ ± 0.75	64.23 ^f^ ± 1.11	66.35 ^f^ ± 1.82	60.64 ^d^ ± 2.31	64.78 ^f^ ± 1.99
Ultrasound-assisted mixed fermentation						
UAP	48.37 ^e^ ± 1.03	71.23 ^a^ ± 0.44	88.39 ^j^ ± 1.53	85.51 ^j^ ± 2.05	86.94 ^j^ ± 0.62	86.11 ^j^ ± 2.12
UAR	43.91 ^c^ ± 0.57	59.67 ^d^ ± 3.99	73.33 ^a^ ± 3.44	77.63 ^b^ ± 0.89	70.17 ^a^ ± 1.34	75.33 ^b^ ± 0.33
UPR	46.84 ^k^ ± 1.08	63.07 ^f^ ± 0.77	68.53 ^l^ ± 3.11	77.39 ^b^ ± 0.16	75.52 ^b^ ± 3.81	75.66 ^b^ ± 0.59

Single culture fermentation (*L. acidophilus* (A), *L. plantarum* (P), *L. reuteri* (R)); mixed culture fermentation (*L. acidophilus*—*L. plantarum* (AP), *L. acidophilus*—*L. reuteri* (AR), and *L. plantarum*—*L. reuteri* (PR)); ultrasound-assisted single fermentation using *L. acidophilus* (UA), *L. plantarum* (UP), *L. reuteri* (UR); ultrasound-assisted mixed fermentation using *L. acidophilus*—*L. plantarum* (UAP), *L. acidophilus*—*L. reuteri* (UAR), and *L. plantarum*—*L. reuteri* (UPR).

## Data Availability

The original contributions presented in this study are included in the article. Further inquiries can be directed to the corresponding author.

## References

[1] Asghari B., Mafakheri S., Zarrabi M.M., Erdem S.A., Orhan I.E., Bahadori M.B. (2019). Therapeutic Target Enzymes Inhibitory Potential, Antioxidant Activity, and Rosmarinic Acid Content of *Echium amoenum*. S. Afr. J. Bot..

[2] Firoznezhad M., Abi-Rached R., Fulgheri F., Aroffu M., Leyva-Jiménez F.J., de la Luz Cádiz Gurrea M., Meloni M.C., Corrias F., Escribano-Ferrer E., Peris J.E. (2023). Design and in Vitro Effectiveness Evaluation of *Echium amoenum* Extract Loaded in Bioadhesive Phospholipid Vesicles Tailored for Mucosal Delivery. Int. J. Pharm..

[3] Jamnani M.J., Holmelid B., Vedeler A., Parsian H.H., Andersen H.L., Fossen T. (2023). Natural Products from Leaves of the Ancient Iranian Medicinal Plant *Echium amoenum* Fisch. & C. A. Mey.. Molecules.

[4] Darijani S., Afsahi M.M., Akhavan H.-R., Goharrizi A.S. (2025). Drying of Borage (*Echium amoenum*) Flowers Extract: Optimization of Encapsulation and Spouted Bed Drying. Appl. Food Res..

[5] Mofidipour M., Fadaei V., Salehifar M. (2025). Inhibition of Acrylamide and α-Amylase and α-Glucosidase Activities in *Echium amoenum* Powder Fortified Biscuits. Food Res. Int..

[6] Cao C., Sun W., Wu J., Zhao M., Su G. (2025). Ultrasound-Assisted Fermentation on Aroma and Umami Enhancement of Soybean Protein Hydrolysates: Machine Learning-Enhanced Flavoromics and Molecular Insights. Innov. Food Sci. Emerg. Technol..

[7] Divan Khosroshahi E., Razavi S.H., Salami M., Ubeyitogullari A. (2025). Food Bioscience Recent Advances in Bioprocessing of Medicinal Plants through Fermentation: A Promising Approach to Maximize Nutritional/Functional Value, Bioactive Potential, and Health Benefits. Food Biosci..

[8] Yu Z., Su Y., Zhang Y., Zhu P., Mei Z., Zhou X., Yu H. (2021). Potential Use of Ultrasound to Promote Fermentation, Maturation, and Properties of Fermented Foods: A Review. Food Chem..

[9] Umego E.C., He R., Huang G., Dai C., Ma H. (2021). Ultrasound-Assisted Fermentation: Mechanisms, Technologies, and Challenges. J. Food Process. Preserv..

[10] Meena L., Malini B., Byresh T.S., Sunil C.K., Rawson A., Venkatachalapathy N. (2024). Ultrasound as a Pre-Treatment in Millet-Based Probiotic Beverage: It’s Effect on Fermentation Kinetics and Beverage Quality. Food Chem. Adv..

[11] Mehran M., Masoum S., Memarzadeh M. (2020). Improvement of Thermal Stability and Antioxidant Activity of Anthocyanins of *Echium amoenum* Petal Using Maltodextrin/Modified Starch Combination as Wall Material. Int. J. Biol. Macromol..

[12] Abed A., Minaiyan M., Ghannadi A., Mahzouni P., Babavalian M.R. (2012). Effect of *Echium amoenum* Fisch. et Mey a Traditional Iranian Herbal Remedy in an Experimental Model of Acute Pancreatitis. ISRN Gastroenterol..

[13] Khosroshahi E.D., Razavi S.H. (2023). Wheat Germ Valorization by Fermentation: A Novel Insight into the Stabilization, Nutritional/Functional Values and Therapeutic Potentials with Emphasis on Anti-Cancer Effects. Trends Food Sci. Technol..

[14] Kucharska E., Grygorcewicz B., Spietelun M., Olszewska P., Bobkowska A., Ryglewicz J., Nowak A., Muzykiewicz-Szymańska A., Kucharski Ł., Pełech R. (2024). Potential Role of Bioactive Compounds: In Vitro Evaluation of the Antioxidant and Antimicrobial Activity of Fermented Milk Thistle. Appl. Sci..

[15] Hussain A., Bose S., Wang J.H., Yadav M.K., Mahajan G.B., Kim H. (2016). Fermentation, a Feasible Strategy for Enhancing Bioactivity of Herbal Medicines. Food Res. Int..

[16] Wang T., Sheng K., Zhang Y., Jin S., Feng L., Wang L. (2024). Metabolomics Analysis Reveals the Effect of Fermentation on the Chemical Composition and Antioxidant Activity of *Paeonia lactiflora* Root. Heliyon.

[17] Fei L., Zhang D., Li Y., Mkunga J.J., Zhang Z., He C., Shan C., Choudhary M.I., Yang X., Cai W. (2025). Metabolomic Insights into Ultrasound-Assisted Fermentation of Grape Juice. Ultrason. Sonochem..

[18] Qiu L., Zhang M., Chang L. (2023). Effects of Lactic Acid Bacteria Fermentation on the Phytochemicals Content, Taste and Aroma of Blended Edible Rose and Shiitake Beverage. Food Chem..

[19] Zheng Z., Wei L., Zhu M., Qian Z., Liu J., Zhang L., Xu Y. (2023). Effect of Lactic Acid Bacteria Co-Fermentation on Antioxidant Activity and Metabolomic Profiles of a Juice Made from Wolfberry and Longan. Food Res. Int..

[20] Peng X.-Y., Wu J.-T., Shao C.-L., Li Z.-Y., Chen M., Wang C.-Y. (2021). Co-Culture: Stimulate the Metabolic Potential and Explore the Molecular Diversity of Natural Products from Microorganisms. Mar. Life Sci. Technol..

[21] Zhang S., Cui S., Li Q., Zheng X., Liu J. (2025). Mechanistic Understanding of Corn Processing By-Products Valorization via Microbial Ultrasound-Assisted Fermentation: Community Succession and Metabolic Changes. Ultrason. Sonochem..

[22] Kuo C.-H., Chen B.-Y., Liu Y.-C., Chang C.-M., Deng T.-S., Chen J.-H., Shieh C.-J. (2013). Optimized Ultrasound-Assisted Extraction of Phenolic Compounds from *Polygonum cuspidatum*. Molecules.

[23] Carrera C., Ruiz-Rodríguez A., Palma M., Barroso C.G. (2012). Ultrasound Assisted Extraction of Phenolic Compounds from Grapes. Anal. Chim. Acta.

[24] Khosroshahi E.D., Razavi S.H., Kaini H., Aghakhani A. (2022). Improvement of Stability and Antioxidant Activity of Wheat Germ by Mixed Fermentation versus Single Fermentation. J. Food Sci. Technol..

[25] Khosroshahi E.D., Razavi S.H., Kiani H., Aghakhani A. (2023). Mixed Fermentation and Electrospray Drying for the Development of a Novel Stabilized Wheat Germ Powder Containing Highly Viable Probiotic Cultures. Food Sci. Nutr..

[26] Castangia I., Fulgheri F., Leyva-Jimenez F.J., Alañón M.E., Cádiz-Gurrea M.d.l.L., Marongiu F., Meloni M.C., Aroffu M., Perra M., Allaw M. (2023). From Grape By-Products to Enriched Yogurt Containing Pomace Extract Loaded in Nanotechnological Nutriosomes Tailored for Promoting Gastro-Intestinal Wellness. Antioxidants.

[27] Khosravi A., Razavi S.H., Castangia I., Manca M.L. (2024). Valorization of Date By-Products: Enhancement of Antioxidant and Antimicrobial Potentials through Fermentation. Antioxidants.

[28] Bahrololoumi S., Khosroshahi E.D., Razavi S.H., Kiani H. (2024). A Novel Approach for Solubility and Bioavailability Enhancement of Canthaxanthin Obtained from *Dietzia natronolimnaea* HS-1 by Canthaxanthin-V-Amylose Complex. Food Bioproc. Tech..

[29] Akpoghelie P.O., Edo G.I., Ali A.B.M., Yousif E., Zainulabdeen K., Owheruo J.O., Isoje E.F., Igbuku U.A., Essaghah A.E.A., Makia R.S. (2025). Lactic Acid Bacteria: Nature, Characterization, Mode of Action, Products and Applications. Process Biochem..

[30] Chen J., Wang Q., Wu Y., Wu Y., Sun Y., Ding Y., Wei Z., Manickam S., Pan S., Yang J. (2023). Ultrasound-Assisted Fermentation of Ginkgo Kernel Juice by *Lactiplantibacillus plantarum*: Microbial Response and Juice Composition Development. Ultrason. Sonochem..

[31] Xing Y., Aweya J.J., Jin R., Lin R., Weng W., Zhang Y., Deng S., Yang S. (2023). Low-Intensity Ultrasound Combines Synergistically with *Lacticaseibacillus paracasei* Fermentation to Enhance Chitin Extraction from Crab Shells. LWT.

[32] Abesinghe A.M.N.L., Vidanarachchi J.K., Islam N., Prakash S., Samita S., Karim M.A. (2025). Optimization of the Ultrasound-Assisted Fermentation Process of Buffalo’s Milk (*Bubalus bubalis*). LWT.

[33] Wu T., Yu X., Hu A., Zhang L., Jin Y., Abid M. (2015). Ultrasonic Disruption of Yeast Cells: Underlying Mechanism and Effects of Processing Parameters. Innov. Food Sci. Emerg. Technol..

[34] Gholamhosseinpour A., Hashemi S.M.B. (2019). Ultrasound Pretreatment of Fermented Milk Containing Probiotic *Lactobacillus plantarum* AF1: Carbohydrate Metabolism and Antioxidant Activity. J. Food Process Eng..

[35] Ojha K.S., Mason T.J., O’Donnell C.P., Kerry J.P., Tiwari B.K. (2017). Ultrasound Technology for Food Fermentation Applications. Ultrason. Sonochem..

[36] Akdeniz V., Akalın A.S. (2022). Recent Advances in Dual Effect of Power Ultrasound to Microorganisms in Dairy Industry: Activation or Inactivation. Crit. Rev. Food Sci. Nutr..

[37] Huang G., Chen S., Dai C., Sun L., Sun W., Tang Y., Xiong F., He R., Ma H. (2017). Effects of Ultrasound on Microbial Growth and Enzyme Activity. Ultrason. Sonochem..

[38] Ercan M., Akbulut M., Çoklar H., Demirci T. (2025). Impacts of Sonication on Fermentation Process and Physicochemical, Microbiological and Sensorial Characteristics of Fermented Black Carrot Juice. Fermentation.

[39] Shokri S., Terefe N.S., Shekarforoush S.S., Hosseinzadeh S. (2021). Ultrasound-Assisted Fermentation for Enhancing Metabolic and Probiotic Activities of *LactoBacillus brevis*. Chem. Eng. Process.-Process Intensif..

[40] Hajar-Azhari S., Daud N., Muhialdin B.J., Joghee N., Kadum H., Meor Hussin A.S. (2023). Lacto-Fermented Garlic Sauce Improved the Quality and Extended the Shelf Life of Lamb Meat under the Chilled Condition. Int. J. Food Microbiol..

[41] Hashemi S.M.B., Jafarpour D., Soto E.R., Barba F.J. (2022). Ultrasound-Assisted Lactic Acid Fermentation of Bakraei (*Citrus reticulata* Cv. Bakraei) Juice: Physicochemical and Bioactive Properties. Fermentation.

[42] Gaur G., Gänzle M.G. (2023). Conversion of (Poly)Phenolic Compounds in Food Fermentations by Lactic Acid Bacteria: Novel Insights into Metabolic Pathways and Functional Metabolites. Curr. Res. Food Sci..

[43] Aznar-Ramos M.J., De Montijo-Prieto S., del Carmen Razola-Díaz M., Martín-García B., Jiménez-Valera M., Ruiz-Bravo A., Verardo V., Gómez-Caravaca A.M. (2025). Evaluation of the Potential to Increase the Recovery of Phenolic Compounds with Antioxidant and Antidiabetic Activities from Mango Leaves by Fermentation Pre-Treatment and Ultrasound-Assisted Extraction via Sonotrode. Ind. Crops Prod..

[44] Mamy D., Boateng I.D., Chen X. (2025). Metabolomic Changes in *Citrus reticulata* Peel after Conventional and Ultrasound-Assisted Solid-State Fermentation with *Aspergillus niger*: A Focus on Flavonoid Metabolism. Food Chem..

[45] Murtaza M.S., Yaqoob S., Mubeen B., Sameen A., Murtaza M.A., Rehman A., Alsulami T., Korma S.A., Khalifa I., Ma Y.K. (2024). Investigating the Triple-Frequency Ultrasound-Assisted Fermented Rice Lees: Impact on Physicochemical, Structural, Morphological, and Metabolic Properties. Ultrason. Sonochem..

[46] Lou K., Zheng Y., Tan X., Wang L., Tong C., Huang S., Cai X., Zhou C., Cao J., Zhang H. (2024). Influence of Sonication-Assisted Fermentation on the Physicochemical Features and Antioxidant Activities of Yogurts Fortified by Polyphenol-Rich Pineapple Peel Powder with Varied Chemical Profiling. Food Res. Int..

[47] Yang J., Gao T., Wang Q., Xu J., Zhou F., Ding Y., Du H., Pan S., Tao Y., Wu Y. (2024). Ultrasound-Assisted Fermentation of *Porphyra yezoensis* Sauce at Different Growth Stages Using *Lactiplantibacillus plantarum*: Metabolic Response and Biological Activity. Ultrason. Sonochem..

[48] Sun L., Liu L., Yang L., Wang Y., Dabbour M., Mintah B.K., He R., Ma H. (2021). Effects of Low-Intensity Ultrasound on the Biomass and Metabolite of *Ganoderma lucidum* in Liquid Fermentation. J. Food Process Eng..

[49] Tang X., Lv H., Wang H., Yang H., Zhang B., Cong L., Li X., Ma M., Xu Y., Zhang L. (2025). Enhancing Antioxidant Activity of Corn Bract and Silk Juices through Biotransformation of Polyphenols by *Lactobacillus paracasei* TJ199 Fermentation. Food Chem. X.

[50] Agatonovic-Kustrin S., Gegechkori V., Kustrin E., Morton D.W. (2024). The Effect of Lactic Acid Fermentation on the Phytochemical Content of Fig Leaf Extracts Compared to Single Solvent and Sequential Solvents Extraction. S. Afr. J. Bot..

[51] Wang L., Zhang J., Zhang W., Lin X., Li C., Wu Z. (2019). Role of Carbohydrate-Cleaving Enzymes in Phenolic Mobilization of Guava Leaves Tea during Solid State Bio-Processing with *Monascus anka* and *Bacillus* sp.. Process Biochem..

[52] Zhang Q., Gu C., Chang H., Zhang W., Ma L., Liu F., Feng Z. (2025). Effects of Single and Co-Cultured Lactic Acid Bacteria on Antioxidant Capacity and Metabolite Profiles during Rambutan Juice Fermentation. Food Chem. X.

[53] Mamy D., Boateng I.D., Chen X. (2025). Ultrasound-Assisted Solid-State Fermentation by *Aspergillus niger* Increased Phenolics and Antioxidants’ Accumulation in *Citrus reticulata* Peels. Food Biosci..

[54] Ruan S., Li Y., Wang Y., Huang S., Luo J., Ma H. (2020). Analysis in Protein Profile, Antioxidant Activity and Structure-Activity Relationship Based on Ultrasound-Assisted Liquid-State Fermentation of Soybean Meal with *Bacillus subtilis*. Ultrason. Sonochem..

[55] Akbari M., Razavi S.H., Khodaiyan F., Blesa J., Esteve M.J. (2023). Fermented Corn Bran: A by-Product with Improved Total Phenolic Content and Antioxidant Activity. LWT.

[56] do Carmo Brito B.d.N., Campos Chisté R., da Silva Pena R., Abreu Gloria M.B., Santos Lopes A. (2017). Bioactive Amines and Phenolic Compounds in Cocoa Beans Are Affected by Fermentation. Food Chem..

[57] Tsui C.Y., Yang C.Y. (2021). Evaluation of Semi-Solid-State Fermentation of *Elaeocarpus serratus* L. Leaves and Black Soymilk by *Lactobacillus plantarum* on Bioactive Compounds and Antioxidant Capacity. Foods.

[58] Adel Pilerood S., Prakash J. (2014). Evaluation of Nutritional Composition and Antioxidant Activity of Borage (*Echium amoenum*) and Valerian (*Valerian officinalis*). J. Food Sci. Technol..

